# Epanorin, a lichen secondary metabolite, inhibits proliferation of MCF-7 breast cancer cells

**DOI:** 10.1186/s40659-019-0261-4

**Published:** 2019-10-10

**Authors:** Juan Palacios-Moreno, Cecilia Rubio, Wanda Quilhot, M. Fernanda Cavieres, Eduardo de la Peña, Natalia V. Quiñones, Hugo Díaz, Flavio Carrión, Carlos F. Henríquez-Roldán, Caroline R. Weinstein-Oppenheimer

**Affiliations:** 1 Escuela de Química y Farmacia, Facultad de Farmacia, Universidad de Valparaíso, Av. Gran Bretaña 1093, Playa Ancha, CP 2360102 Valparaiso, Chile; 2 Centro de Investigación Farmacopea Chilena, Valparaiso, Chile; 3 ICA-Mutagénesis Ambiental, Consejo Superior de Investigaciones Científicas, Madrid, Spain; 4 Escuela de Ingeniería en Medioambiente, Facultad de Ingeniería, Universidad de Valparaíso, Valparaiso, Chile; 5 Programa de Inmunología Traslacional, Facultad de Medicina, Clínica Alemana, Universidad del Desarrollo, Las Condes, Santiago, Chile; 6 Instituto de Estadística, Facultad de Ciencias, Universidad de Valparaíso, Playa Ancha, Valparaiso, Chile

**Keywords:** Epanorin, Cancer, Cytotoxicity, Mutagenesis, Cell cycle, Apoptosis

## Abstract

**Background:**

Epanorin (EP) is a secondary metabolite of the *Acarospora* lichenic species. EP has been found in lichenic extracts with antimicrobial activity, and UV-absorption properties have been described for closely related molecules; however, its antiproliferative activity in cancer cells has not yet been explored. It has been hypothesized that EP inhibits cancer cell growth. MCF-7 breast cancer cells, normal fibroblasts, and the non-transformed HEK-293 cell line were exposed to increasing concentrations of EP, and proliferation was assessed by the sulforhodamine-B assay.

**Results:**

MCF-7 cells exposed to EP were examined for cell cycle progression using flow cytometry, and DNA fragmentation was examined using the TUNEL assay. In addition, EP’s mutagenic activity was assessed using the *Salmonella typhimurium* reverse mutation assay. The data showed that EP inhibits proliferation of MCF-7 cells, and it induces cell cycle arrest in G0/G1 through a DNA fragmentation-independent mechanism. Furthermore, EP’s lack of overt cytotoxicity in the normal cell line HEK-293 and human fibroblasts in cell culture is supported by the absence of mutagenic activity of EP.

**Conclusion:**

EP emerges as a suitable molecule for further studies as a potential antineoplastic agent.

## Background

Cancer incidence is increasing worldwide with a projected rise in new cases from 18.1 million in 2018 to 29.4 million in 2040, becoming the first or second cause of death before the age of 70 in more than 50% of countries [13]. The most frequent type of cancer among women is breast cancer [7] with an estimated 60–80% being estrogen receptor alpha positive [26].

The contribution of natural origin products as anticancer and chemopreventive agents is widely recognized, as it is also well-established that there is a great need for new molecule development with fewer side effects [2, 10]. Cell lines have been an extensively used tool for the screening of potential new chemical entities to treat cancer. In this study, the MCF-7 breast cancer cell line was used as a model to explore EP as a new antiproliferative molecule because it represents a traditional and widely studied estrogen receptor alpha positive cancer that has shown to be suitable for screening anticancer drugs [6].

Lichens are symbiotic organisms consisting of a fungus and a photosynthetic partner. Their capacity to produce and accumulate secondary metabolites gives rise to their wide chemical diversity which includes over 1000 compounds. Many of these substances are unique to lichen fungi and may be synthesized as stress metabolites, metal detoxifiers or for protection against predators, pathogens or UV radiation. Some of these metabolites also exhibit in vitro biological activities on prokaryotic and eukaryotic cell models, including antimicrobial, antioxidant and antiproliferative properties [5, 17, 24] which make them potential candidates as human therapeutic agents.

**Fig. 1:**
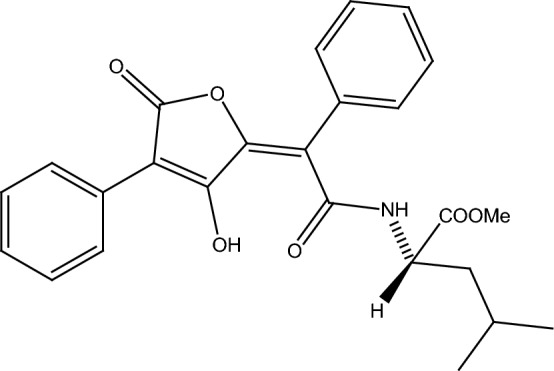
Structure of Epanorin (Huneck). A yellow solid shikimic acid-derived metabolite extracted form *Acarospora schleicheri* A. Massal

EP (Fig. 1) is a shikimic acid-derived metabolite found, among others, in Acarospora species. In the lichen, it might be a photoprotector due to its structural similarity to other pulvinic acid derivatives that absorb UV radiation [11, 15], and it has also been shown to be present in an extract that inhibits growth of several Gram positive bacteria [32]. In a routine screening for biological activity of several lichenic metabolites, EP inhibited MCF-7 cell viability. Here, the analysis aimed to gain basic understanding about the antiproliferative activities of this natural origin molecule is reported. Flow cytometry was used to evaluate cell cycle progression and TUNEL assay for detection of DNA fragmentation as a marker for apoptosis induction in cells cultured in the presence of EP. Also, mitochondrial reactive oxygen species (ROS) production within EP-exposed cells was assessed by means of a specific fluorescent probe. Additionally, the *Salmonella typhimurium* reverse mutation assay was employed to evaluate mutagenic properties of EP.

## Methods

### Botanical material

EP (methyl (2S)-2-[[(2Z)-2-(3-hydroxy-5-oxo-4-phenylfuran-2-ylidene)-2-phenylacetyl] amino]-4-methylpentanoate) was isolated from *Acarospora schleicheri* A. Massal, and collected in altitudinal gradients in Enquelga-Isluga (19°14′S, 68°47′W) in Chile´s alpine zones. In each site, at least ten thalli were randomly collected from rock surfaces. Voucher specimens were deposited in the Lichen Herbarium of the School of Chemistry and Pharmacy, Universidad de Valparaíso. The collection of the specimens was authorized by the National Forest Corporation (CONAF).

### EP extraction

Thalli were cleaned and washed with distilled water, and dried at 60 °C. EP was extracted in acetone at room temperature (20 °C ± 2°) for 48 and 24 h successively. The extract was then purified chromatographically using a silica gel Merck 60 G (0.032–0.063 nm) column eluted with a mixture of hexane and ethyl-acetate with increasing polarity. The fractions were monitored by thin-layer chromatography (TLC) using silica gel Merck 60 F_254_ plates. The blots were developed using a H_2_SO_4_ spray reagent and UV light (254/365 nm).

### EP identification

RMN spectroscopic analysis was utilized. The ^1^H- and ^13^C-NMR spectra were recorded in CDCl_3_ solutions and are referenced to the residual peaks of CHCl_3_ at δ = 7.26 ppm and δ = 77.00 ppm for ^1^H and ^13^C on an Avance 400 Digital NMR spectrometer (Bruker, Rheinstetten, Germany) operating at 400.1 MHz for ^1^H and 100.6 MHz for ^13^C.

Optical rotation was measured with a sodium lamp (λ = 589 nm, D line) on a Atago AP-300 digital polarimeter equipped with 1 dm cells at 23 °C.

### Cell culture

The human breast adenocarcinoma cell line MCF-7 (American Type Culture Collection, (ATCC^®^ HTB-22™), Rockville, MD, USA) and the human epithelial kidney HEK293 cells (ATCC^®^ CRL-11268™) were grown in DMEM (Invitrogen, Carlsbad, CA, USA), supplemented with 10% fetal bovine serum (FBS, PAA Laboratories GmbH, Linz, Austria), 2 mM glutamine, 10 U/L penicillin and 100 μg/mL streptomycin (Thermo Fischer Scientific, Waltham, MA USA). The cells were cultured in an incubator (Thermo Forma) with a 5% CO_2_ humidified atmosphere.

### Cell proliferation assay

Cells were seeded into 96-well cell culture plates at a density of 5 × 10^3^ cells/well. After 24 h incubation, cells were exposed for 48 h to 14, 28, 42, 56, 70, 84 and 98 μM EP in dimethyl sulfoxide (DMSO). Equivalent concentrations of DMSO vehicle, corresponding to the different dilutions of the test metabolite, and cells without treatment were included as negative controls. Cell proliferation inhibition by 1.3 μM tamoxifen (TMX) was used as positive control. Cell proliferation was determined with sulforhodamine-B (SRB, Sigma Aldrich, St Louis, MI) assay [30]. At the end of the culture period, proteins were precipitated with 50% w/v trichloroacetic acid and cells were stained with 50 μL of SRB (0.4% w/v in 1% v/v acetic acid). Finally, 200 μL 10 mM tris(hydroxymethyl) aminomethane (TRIS) were added to each well and absorbance was read at 540 nm using a microplate reader (Merck Sensident Scan).

### DNA fragmentation assay

Detection of DNA fragmentation as indicator of apoptosis was performed by the in Situ Cell Death Detection Kit (TUNEL Kit, Roche Applied Science, Manheim, Germany) [19]. MCF-7 cells were grown on silanized slides until 40% confluence. Then, the cells were treated for 12 h with 28 μM EP in DMSO, and 50 μM TMX, DMSO, and untreated cells as positive, vehicle and negative controls, respectively. At the end of the exposure, cells were washed five times with phosphate saline buffer (PBS) and fixed 20 min with 2% p-formaldehyde at 4 °C. After washing them five times with PBS, apoptosis was determined following manufacturer’s instructions, adding 4’,6-diamidino-2-phenylindole (DAPI) to stain the nuclei and using a technical negative control with a slide to which no terminal deoxynucleotidyl transferase (TdT) was added. Images were visualized with an Olympus BX 51 fluorescence microscope provided with a U-MWU2 Olympus filter.

### Cell cycle analysis by flow cytometry

This protocol was performed by adapting the report of Riccardi and Nicoletti [28]. For this, 20 × 10^4^ cells were seeded in cell culture flasks and incubated for 24 h, after which they were treated for 48 h with 28 μM EP, 1.3 μM TMX, DMSO, and untreated cells were used as positive, vehicle, and negative controls, respectively. Cells were then trypsinized, aliquoted into flow cytometry tubes (1 × 10^6^ cells/tube) and centrifuged 5 min at 240*g*. The pellet was resuspended into 500 μL PBS, and kept in ethanol at 4 °C until its analysis, at which time cells were centrifuged for 5 min at 240*g*, pellets were washed with 1 mL 10% p/v albumin in PBS, centrifuged for 5 min at 240*g*, and resuspended with 500 μL Krisham solution (1.12 mg/mL sodium citrate, 0.046 mg/mL propidium iodide, 0.01% v/v triton X-100 and 0.01 mg/mL RNAase A) for DNA staining. DNA fluorescence was detected with a flow cytometer [Coulter (R) Epics (R)]. For cell counting, a minimum of 3500 events were recorded for each treatment. Fluorescence intensity histograms versus event numbers were recorded.

### Mitochondrial oxygen reactive species assay

The mitoSOX red fluorescent probe was utilized for the assessment of mitochondrial derived oxygen reactive species [34]. The assay was performed seeding 5000 MCF-7 cells on a 96-wells culture plate. After 24 h, the cells were exposed to 1 µM doxorubicin (positive control), 28 μM EP or 1% DMSO containing cell culture media for 48 h. Then, the cells were washed twice with PBS and exposed to mytoSOX for 10 min; next, they were washed twice again with PBS and the fluorescence detected at 510/595 nm of excitation and emission, respectively, with a Varioscan™LUX multimode plate reader (Thermoscientific). Next, the protein content was determined by using the sulforhodamine B (SRB) assay. For this, the cells were fixed using cold 1% acetic acid in methanol and then exposed to SRB 0.5% w/v for 1 h at 37 °C. After removing the SRB, the wells were gently washed with 1% acetic acid, then the plate was dried and the fixed dye solubilized by adding 200 μL of 10 mM tris pH 10. The absorbance was read with the Varioscan™LUX multimode plate reader (Thermoscientific) at 580 nm. The results were expressed as relative fluorescence units normalized by the protein content of each well.

### Mutagenicity evaluation

Testing was performed according to standard *S. typhimurium* reverse mutation assay (Ames test) procedures [1, 21]. In brief, four different histidine-deficient (his−) *S. typhimurium* strains TA98, TA100, TA102 and TA 104 were used. For metabolic activation, S9 fraction was obtained from the supernatant of post-mitochondrial mouse liver fraction exposed to sodium phenobarbital and β-naphtoflavone [9]. E was dissolved in DMSO and tested at 0.125, 12.5 and 125 μg/plate. Each strain was incubated in the presence of E at 37 °C for 48 h with and without metabolic activation. Spontaneous reversions and mutations in response to DMSO and known mutagens (4-nitroquinoline-*N*-oxide for TA98, methylmethanesulfonate for TA 100 and TA102 and methylglyoxal for TA104) were also determined for negative and positive controls, respectively. Each treatment was performed in triplicate.

### Statistical analysis

For proliferation assays, 42 independent experiments were conducted. The percentage of inhibition for E was expressed as π (%iEX) with the following statistical hypothesis: H_0_: π (%iEX) = 0 and H_1_: π (%iEX) > 0. The results of the assay gave an estimation of π (%iEX) which was called p (%iARX), and a function of this sample proportion allowed to analyze the hypothesis. The Stata software was used [31] to calculate the signification probability, which allowed to reject H_0_ when it was below 0.05.

## Results

### EP identification

**Fig. 2:**
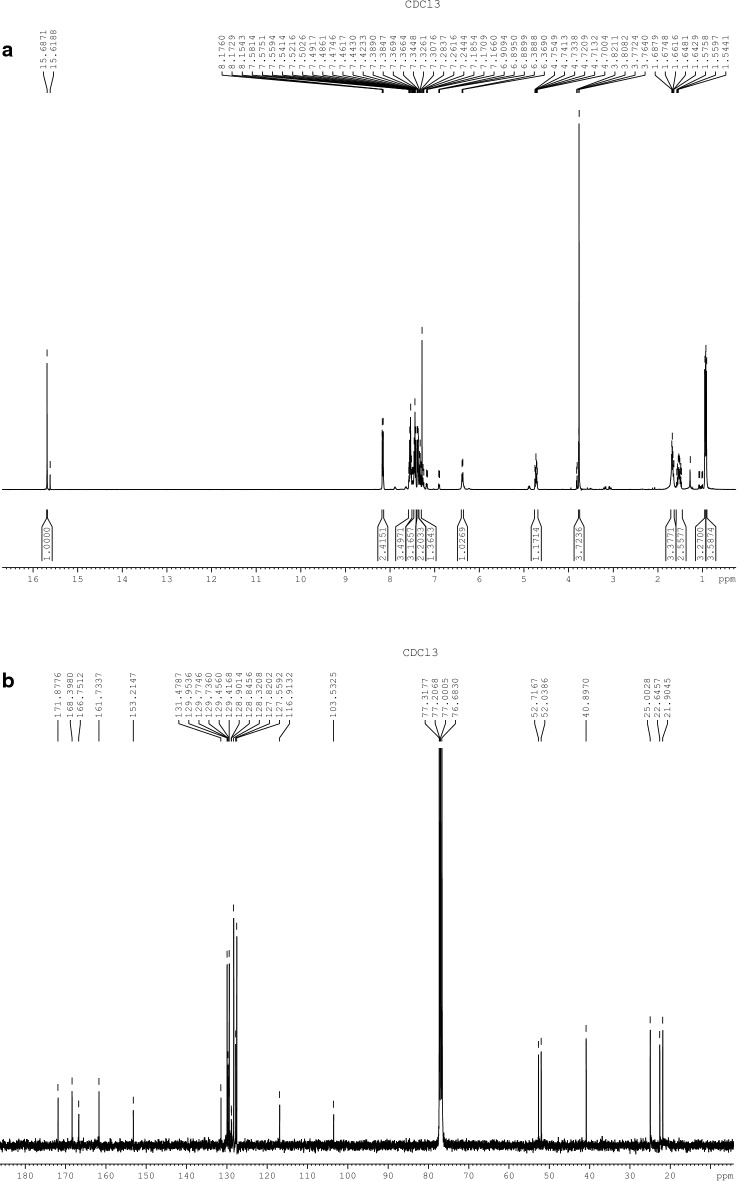
^1^H-RMN (**a**) and ^13^C-RMN (**b**) spectra for EP. The assignment of EP was according to the following: ^1^H-RMN (CDCl_3_) δ: 15.7 (1H, s, OH); 8.2 (1H, d, *J *= 1.2 Hz NH); 7.6–7.3 (10H, m, H–Ar); 4.8–4.7 (1H, m, NHCH); 3.8 (1H, s, O–CH_3_); 1.7–1.6 (3H, m, CH_2_ CH(CH_3_)_2_); 0.9 (3H, d, *J *= 6.32 Hz, CH(CH_3_)_2_), 0.9 (3H, d, *J *= 6.32 Hz, CH(CH_3_)_2_) and ^13^C-RMN (CDCl_3_) δ: 171.9 (CONH); 168.4 (COOCH_3_); 166.5 (COO); 161.7 (COH); 153.2 (OCCOH); 130.0 (C–Ar); 129.8 (C–Ar); 129.7 (C–Ar); 129.5 (C–Ar); 129.4 (C–Ar); 128.9 (C–Ar); 128.8 (C–Ar); 128.3 (C–Ar); 127.8 (C–Ar); 127.6 (C–Ar); 116.9 (COC-Ar); 103.5 (COCOH); 52.7 (O-CH_3_); 52.0 (NHCH); 40.9 (CH_2_); 25.0 (CH(CH_3_)_2_; 22.6 (CH(CH_3_)_2_; 21.9 (CH(CH_3_)_2_

EP was isolated as a yellow solid (m.p. 134.5 °C). Structural confirmation of EP (shown in Fig. 1) was based on an analysis of ^1^H and ^13^C NMR spectra presented in Fig. 2a, b, respectively. Specifically, the ^1^H NMR spectrum, showed signals at δH = 15.7 ppm (1H, s) assigned to hydrogen of OH. At δH = 7.6–7.3 ppm the presence of ten aromatic protons is observed, and at δH = 0.9 ppm two signals were observed (3H, d, J = 6.32 Hz), which were assigned to methyl of isopropyl group. On the other hand, the ^13^C NMR confirmed the presence of three carbonyls by their chemical shifts at 171.9, 168.4 and 166.5 ppm. Additionally, ten aromatic carbons at δ 130.0 and 127.6 ppm were evident. Finally, the presence of aliphatic carbons was confirmed by their chemical shifts at 40.9, 25.0, 22.6 and 21.9 ppm.

The assignment of EP was according to the following:

^1^H-RMN (CDCl_3_) δ: 15.7 (1H, s, OH); 8.2 (1H, d, *J *= 1.2 Hz NH); 7.6–7.3 (10H, m, H–Ar); 4.8–4.7 (1H, m, NHCH); 3.8 (1H, s, O–CH_3_); 1.7–1.6 (3H, m, CH_2_ CH(CH_3_)_2_); 0.9 (3H, d, *J *= 6.32 Hz, CH(CH_3_)_2_), 0.9 (3H, d, *J *= 6.32 Hz, CH(CH_3_)_2_).

^13^C-RMN (CDCl_3_) δ: 171.9 (CONH); 168.4 (COOCH_3_); 166.5 (COO); 161.7 (COH); 153.2 (OCCOH); 130.0 (C–Ar); 129.8 (C–Ar); 129.7 (C–Ar); 129.5 (C–Ar); 129.4 (C–Ar); 128.9 (C–Ar); 128.8 (C–Ar); 128.3 (C–Ar); 127.8 (C–Ar); 127.6 (C–Ar); 116.9 (COC–Ar); 103.5 (COCOH); 52.7 (O–CH_3_); 52.0 (NHCH); 40.9 (CH_2_); 25.0 (CH(CH_3_)_2_; 22.6 (CH(CH_3_)_2_; 21.9 (CH(CH_3_)_2_.

In addition to the spectral data, the optical rotation gave a value of [α]_D_ = − 1.81° (CHCl_3_, c = 0.59), confirming EP identity according to Huneck and Yoshimura [12].

### Selective proliferation inhibition by EP

**Fig. 3:**
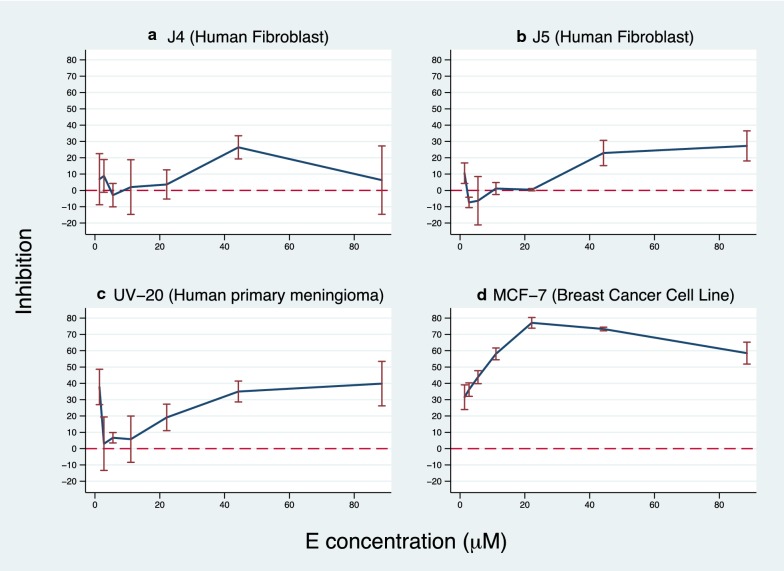
Sulforhodamine B proliferation assays. The inhibition of EP on cell proliferation was tested for normal human fibroblasts (**b**, **c**), a primary culture of menangioma (**a**) and the MCF-7 cell line (**d**). Cells were cultured for 48 h in the presence or absence of EP at concentrations from 14 to 98 μM. After quantifying proteins at each well by the sulforhodamine assay, the percentage of inhibition was calculated from the ratio of the signals with each tested concentration of EP and the cells grown in cell culture media. This is the result of a single experiment performed in triplicates

EP’s capacity to inhibit cell proliferation was tested on the MCF-7 cell line, and on primary cultures of menangioma and fibroblasts. As seen in Fig. 3, the inhibitory activity was outstanding for the breast cancer cell line (up to 80%), relevant for the meningioma (up to 40%) and irrelevant for most of the tested concentrations for the HEK293 normal cell line and human fibroblasts (up to 20%).

**Fig. 4:**
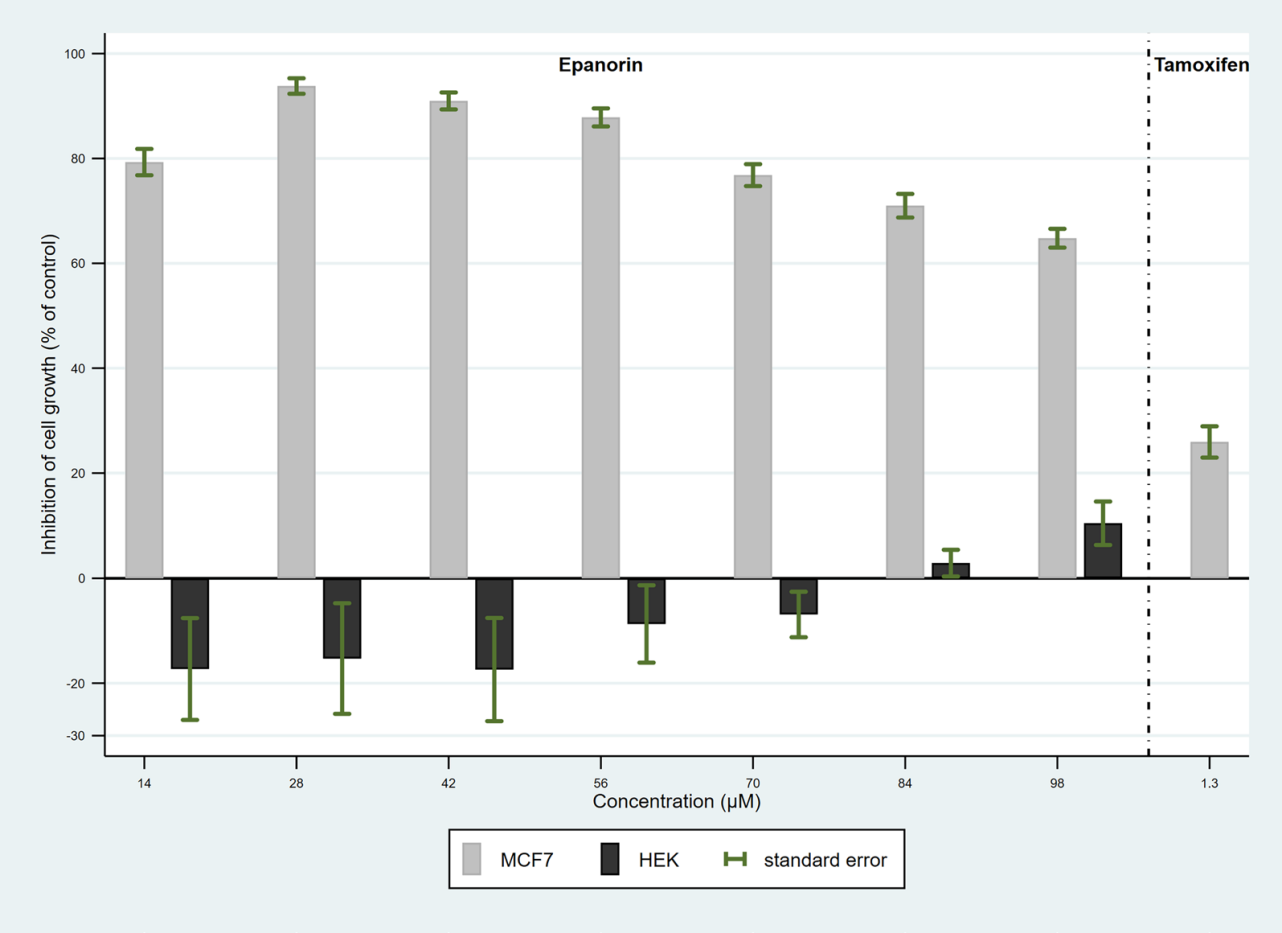
Dose response analysis for the inhibition of MCF-7 and HEK293 cells proliferation by EP and TMX. Cells were cultured for 48 h in the presence or absence of EP (14 to 98 µM) or TMX (1.3 µM) for MCF-7 and EP (14 to 98 µM) for HEP293 cells. The percentage of inhibition was calculated from the ratio of the signals in both conditions. The gray bars represent the results of 42 independent experiments performed with triplicates for the MCF-7 cells, and the black bars show the results for one experiment performed with triplicates for the HEP293 cell line. The standard error is shown in each bar

Next, as positive control, a dose response experiment was conducted with 42 replicates to establish confidence intervals for proliferation inhibition by EP and tamoxifen (TMX) on the MCF-7 cells (Fig. 4). The TMX concentration was chosen according to the estimated plasma levels achievable in a patient after an oral dose. The inhibition of proliferation for EP at 28 µM was the highest and resulted statistically significant (p < 0.001), therefore, next experiments were conducted at this concentration.

**Fig. 5:**
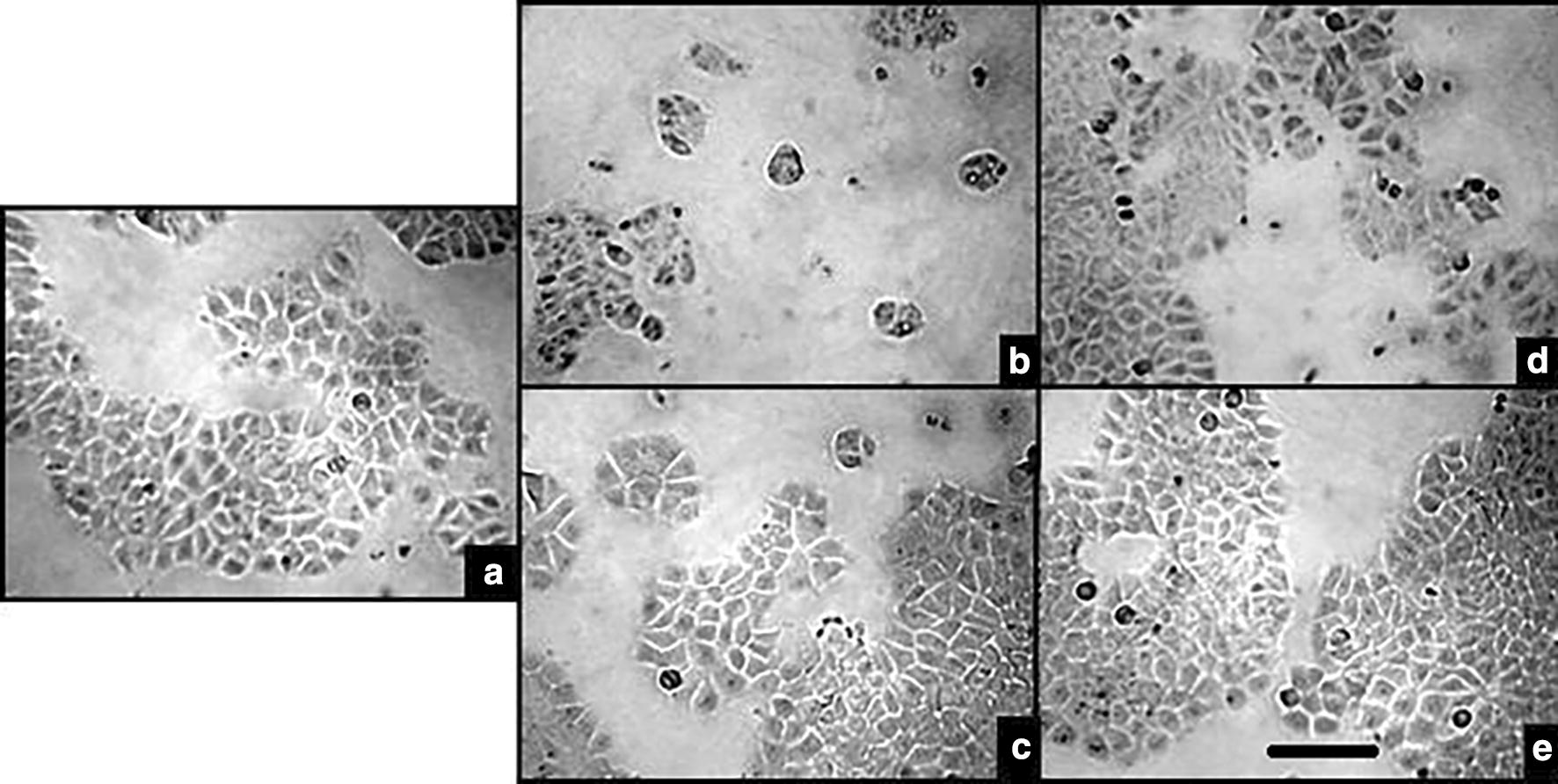
Optical microscopy photography. Taken at 40X for MCF-7 for cells grown in cell culture media (**a**), 28 µM EP (**b**), its DMSO solvent control (**c**), 1.3 µM TMX (**d**) and its ethanol solvent control (**e**). There are no noticeable differences among cells grown in drug free cell culture media, DMSO or ethanol containing cell culture media. Cells grown in EP or TMX show a reduction in the number of colonies. Bar scale: 100 µm

In addition, the visible changes of the cell cultures exposed to EP or TMX were documented by optical microscopy, showing a noticeable reduction in cell colonies specially with the EP exposed cultures (Fig. 5). This was not seen in the respective solvent control (same DMSO concentration) cell culture.

### EP induced oxidative stress assay

**Fig. 6:**
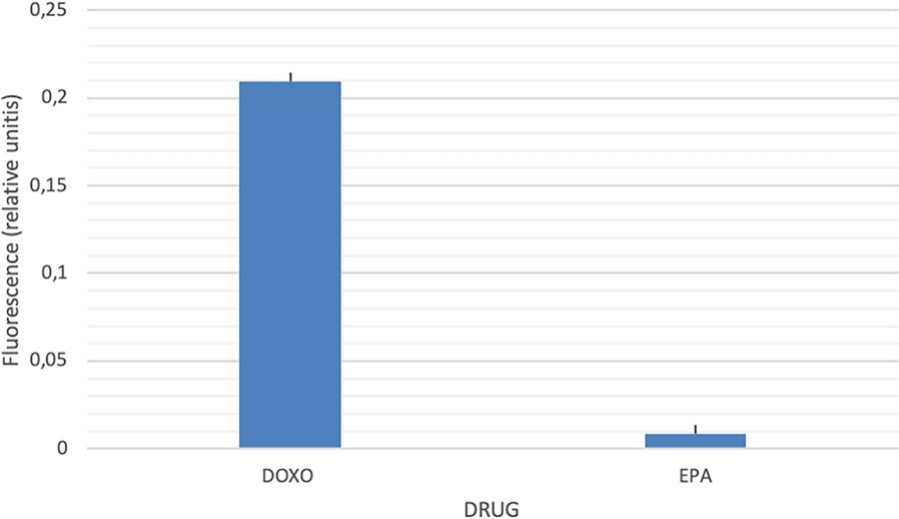
MitoSOX mitochondrial oxygen reactive species assay. The assay was performed on 5000 cells exposed to 28 μM EP, 1 µM doxorubicin or DMSO control for 48 h. The cells were then exposed to mytoSOX and fluorescence was read at 510/595 nm excitation/emission. The results were normalized by the content of protein in each well, determined by sulforhodamine. The bars represent the mean values of the normalized fluorescence and standard error is shown

MitoSOX was employed as a probe to detect mitochondrial oxidative stress, the most relevant source of ROS. Doxorubicin is recognized for its capacity to induce oxidative stress, therefore it was utilized as a positive control. As seen in Fig. 6, non detectable mitochondrial ROS were found in MCF-7 cells exposed to EP as compared to doxorubicin incubated MCF-7 cells.

### Cell cycle effect of EP

**Fig. 7:**
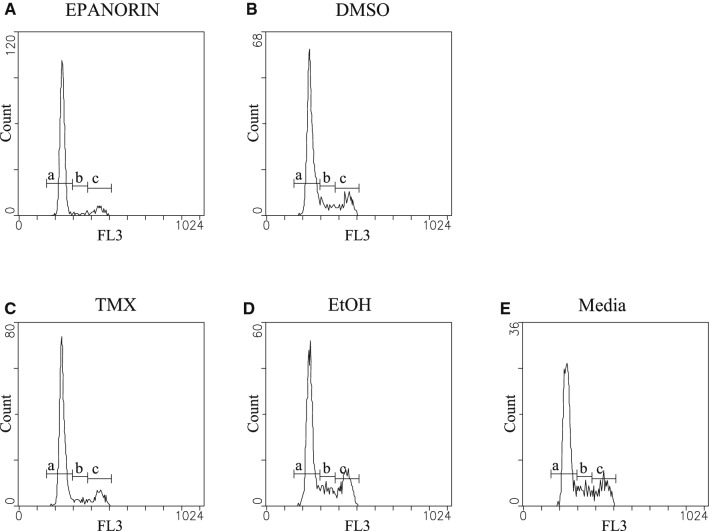
Histograms for cell cycle analysis by flow cytometry on MCF-7 cells. Treatment were 28 µM E (**A**), DMSO (**B**) 1.3 µM TMX (**C**), ethanol (**D**) or non-exposed cultures (**E**). a, b and c represent cells in Go/G1, S and G2M, respectively and FL3 represents the popidium iodide fluorescence. Letter a corresponds to Go, b to S and c to G_2_M phases of the cell cycle. This figure is representative of two independent experiments and it shows that solvent treated cells behave like cells grown in cell culture as opposed to EP and TXM exposed cells that exhibit cell cycle arrest

EP induced a Go cell cycle arrest with 86% of the cells on this phase of the cell cycle, opposed to 73% on its solvent control (DMSO). The percentage of cells on the S phase of the cell cycle diminished from 10% on the control to 4.3 for the EP exposed cell culture. The TXM treated cells, used as positive control, also exhibited a Go arrest, showing a 79% of cells in this phase, compared with 65% for its solvent control (ethanol). Regarding the percentage of cells within the S phase, it decreased from 13% for the control cells to 7% for the tamoxifen treated cells. Both molecules exhibited similar percentages of change among cells in Go and S phases in relation to their respective solvent control. The histograms that represent the cell cycle progression for all the experimental conditions are shown in Fig. 7.

### DNA-fragmentation in EP exposed MCF-7 cells

**Fig. 8:**
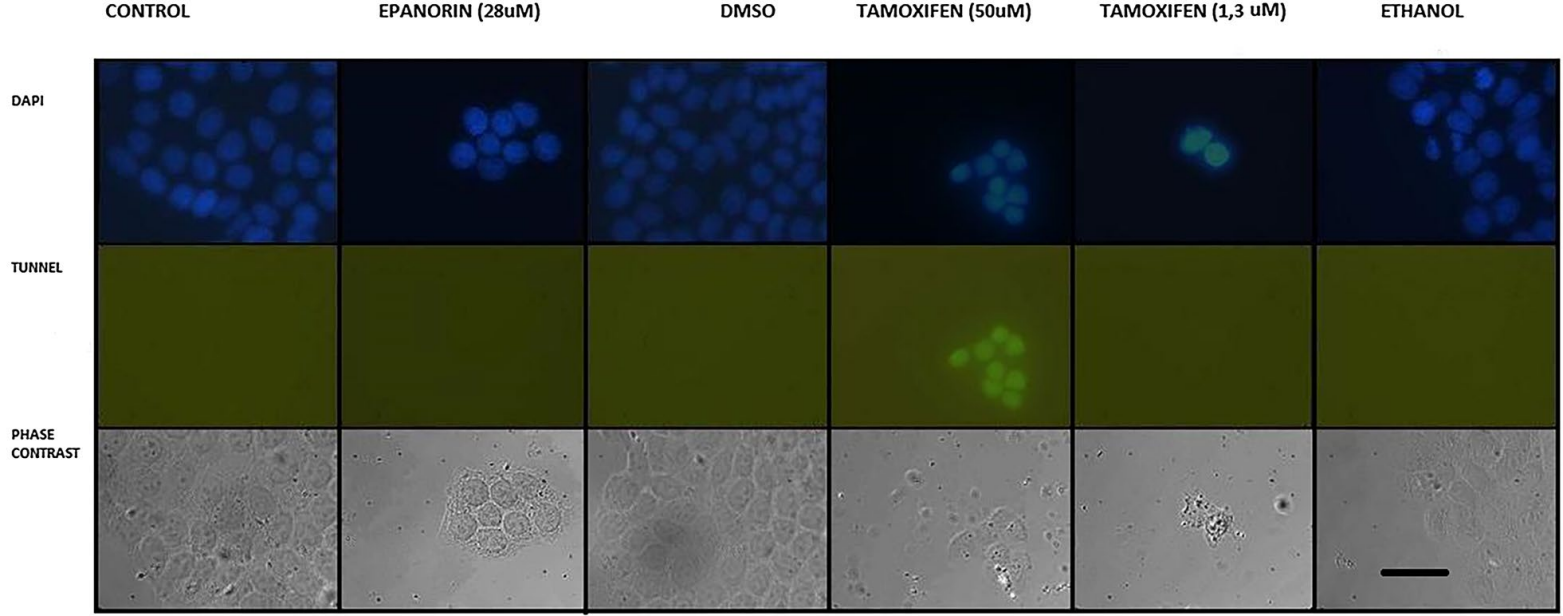
Apoptosis determination by tunnel assay. The cells were exposed to media with no additions (CONTROL), 28 µM E and its solvent control DMSO, 50 µM TMX, and 1.3 µM TMX and ethanol as their solvent control. This image represents two independent experiments. The first raw represents nuclei stained with DAPI, the second, the tunnel assay, and the third, the cells observed by phase contrast microscopy. The nuclei and phase contrast observations show that EP and TXM are reducing the number of cells in culture. Only 50 µM TMX gives a positive nuclear green fluorescent signal consistent with DNA fragmentation. Bar scale: 100 µm

The cells were cultivated during 12 h at the experimental conditions and then the tunnel assay was performed to detect DNA fragmentation. As presented in Fig. 8, EP did not induce detectable DNA fragmentation, as no green fluorescence was observed. Also, TXM at the previous tested conditions, 1.3 µM did not show any signals of DNA fragmentation. To check for the assay performance a 50 µM concentration was assayed and a positive nuclear fluorescent signal was observed, which is consistent with DNA fragmentation (Fig. 8).

### Mutagenicity of EP

Table 1 shows the number of revertants after culturing TA98, TA100, TA102 and TA104 Salmonella strains with increasing doses of EP. EP did not increase the number of colonies of any of the strains in the presence or in the absence of the S9 metabolic activation system. On the contrary, all standard mutagens, used as positive controls for the validation of the test conditions, caused an increase in the number of revertant colonies, further indicating that EP is not mutagenic in this assay.

**Table 1:** Number of revertants in four different *Salmonella typhimurium* strains incubated with increasing concentrations of etanorin

Treatment	Number of histidine + revertants							
	TA98		TA100		TA102		TA104	
	S9−	S9+	S9−	S9+	S9−	S9+	S9−	S9+
Spontaneous revertions	30 ± 2	22 ± 4	40 ± 7	38 ± 6	345 ± 43	328 ± 28	315 ± 23	365 ± 48
DMSO	26 ± 6	24 ± 3	42 ± 3	39 ± 7	305 ± 32	324 ± 35	363 ± 32	321 ± 24
Standard mutagens^a^	87 ± 14	968 ± 192	320 ± 66	790 ± 59	1416 ± 34	4848 ± 113	2069 ± 125	4625 ± 167
E 0.125 µg/plate	26 ± 6	29 ± 2	52 ± 5	56 ± 6	352 ± 25	359 ± 9	384 ± 46	361 ± 52
E 12.5 µg/plate	26 ± 4	27 ± 1	50 ± 4	40 ± 3	306 ± 19	352 ± 21	325 ± 41	374 ± 28
E 125 µg/plate	22 ± 6	22 ± 2	47 ± 9	40 ± 6	296 ± 47	340 ± 21	359 ± 36	346 ± 44

^a^Standard mutagens: 4-nitroquinoline-*N*-oxide for TA98, methylmethanesulfonate for TA100 and TA102 and methylglyoxal for TA104

## Discussion

Lichenic extracts and their secondary metabolites have been extensively studied for their pharmacologic activity as antibiotics, antiparasitic, anti fungal, antioxidants, antiproliferative agents, among others [18, 27, 32]. However, EP bioactivity has not yet been examined, with the exception of a report that showed its presence in a methanolic extract with antimicrobial activity [32]. Herein evidence is provided for the first time for EP inhibition of proliferation of cancer cells with special efficacy on the MCF-7 cancer cells, decreasing its viability by a DNA fragmentation-independent mechanism suggesting that EP does not induce apoptosis. ROS were not detected in EP treated MCF-7 cells, consistent with reports that showed that apoptosis is accompanied with oxidative stress [35]. The lack of mutagenic activity further supports that apoptosis is not a mechanism for the inhibition of MCF-7 cells growth, since mutated cells will normally go into apoptosis. Moreover, ROS were not detected on cells exposed to EP, which is also consistent with EP’s absence of mutagenic activity, since ROS are recognized for their mutagenic activity [16, 36].

On the other hand, there is a good correlation between the mutagenic and carcinogenic activity of a molecule [22] which is explained by the fact that some carcinogens are mutagens and that certain types of cancers are produced as a result of somatic mutations. Maron and Ames [21] proposed the *S. typhimurium* reverse mutation assay which has become an efficient technique to detect potential mutagens. Positive results in this assay are an indication that further mammalian evaluations must be performed, while lack of activity in the Ames system, such as the one reported here for EP, is indicative that the molecule may not pose a carcinogenicity risk, at least not through a mutation-mediated mechanism. This is an important property for novel antineoplastic agents, since conventional ones that act at the DNA level (i.e. anti metabolites or alkylating agents) are mutagenic, being this a dangerous side effect of anti-cancer treatments.

The mechanisms of EP cytotoxicity do not involve DNA fragmentation since doses of the molecule that significantly reduced cell viability, failed to give tunel positive cells. This could be explained because MCF-7 cells are resistant to apoptosis which has been attributed to their functional deletion of caspase 3, a key serine protease for this cell death pathway [14]. However, McGee et al. [23] showed apoptosis and DNA fragmentation of MCF-7 cells exposed to a pyrrolo-1,5-benzoxazepine; nevertheless, the percentages of cells undergoing apoptosis were only slightly above 30. This supporting that, independent of the mechanism of cell death, MCF-7 cells might be considered death-resistant, which highlights the excellent performance of EP as a cytotoxic agent that exhibited, at the most effective concentration, 90% of cell growth inhibition. Moreover, at the same inhibitory concentration (28 µM) EP exhibited neglectable inhibition on non-transformed fibroblasts. And inhibition of the HEP293 human normal cells was modest (below 20%), which makes this molecule an interesting candidate for further studies for a novel antineoplastic agent with less side effects on bystander cells.

ROS were analyzed through the mytoSOX probe because p53-mediated cell cycle arrest in MCF-7 cells associated with mitochondrial apoptosis has been reported [36]. However, no ROS production was detected in the MCF-7 cells exposed to EP, which is consistent with a previous report that showed ROS-independent cell cycle arrest [37]. EP exhibited a G1 cell cycle arrest which was similar in magnitude to that shown by TXM, a clinically used anti proliferative drug for estrogen positive breast cancer cells. Moreover, the effects on cell cycle progression were comparable to the reported for coumestrol on the MCF-7 cells [37]. On the other hand, the effect of EP on cell cycle arrest is significant, since pharmacologists have seen an opportunity in the examination of cell cycle phases for anticancer drug discovery which recently resulted in a hit to the market of CDK’s inhibitors [25, 33]. Herein EP showed a G0/G1 cell cycle arrest effect which explains, at least partially, the results of EP inhibition on the cell growth assay (sulforhodamine B). A recent report focused on the search of inhibitors of the cell cycle phases, rendered 69 G1-phase inhibitors, 148 S-phase inhibitors, and 273 G2/M-phase inhibitors. So, apparently, there is less representation of G1 inhibitors on common libraries of small molecule inhibitors, which makes this result a contribution to this less represented group, and it could give structural information for both basic and clinical oriented research [29]. EP inhibited cell cycle with 86% of the cells at Go/G1 as compared with the best G1 inhibitor found by Senese et al. [29], staurosporine, with a 86.65 of the cells at this stage. Since staurosporine is a known pKC inhibitor, it could be interesting to test EP’s capacity to inhibit kinases that are critical for cell cycle progression.

Bačkorová et al. [4] have recently investigated the mechanism of cytotoxicity of four lichen secondary metabolites (parietin, atranorin, usnic acid and gyrophoric acid) on HT-29 cancer cell line. They found that usnic acid and atranorin were capable of inducing apoptosis via a caspase-3 mechanism activation. They also detected a series of proteins (PARP, p53, Bcl-2/Bcl-xL, Bax, p38, pp38) expressed by the cells which further evidence apoptosis induction. The different results with respect to this report might be attributed to structural issues, since usnic acid is a dibenzophenone, atranorin is a depside, and EP is a pulvinic acid derivative. Unfortunately, the pulvinic acid derivatives have not been analyzed for antiproliferative activity. In consequence, a comparison of EP with them is not plausible. Interestingly, these compounds have shown anti-inflammatory activity [8], but mechanisms have not been evaluated. Anti-inflammatory activity might be associated with antiproliferative effects [20], thus exploring this avenue in future research could be worthwhile.

Therefore, these molecules probably have different cell targets, which requires further research. On the other hand, different cancer cells have also shown to have differentiated responses to the same lichenic compound [3] so it is possible that EP could induce apoptosis on cells other than MCF-7. This could be the case, since MCF-7 cells have been shown to lack caspase 3, as it was explained above, which would explain resistance to apoptosis. This makes the results reported herein especially valuable since a molecule is shown which is suitable for killing more aggressive cancers which are apoptosis-resistant and only modestly sensitive to a conventional anti-cancer agent as tamoxifen used for estrogen receptor positive breast cancers. Further studies are necessary to fully understand the antiproliferative mechanisms of EP.

## Conclusion

EP, a non-mutagenic molecule recovered from *Acarospora schleicheri* A. Massal, has the potential to become a novel anti-breast cancer drug based on its capacity to inhibit the proliferation of a breast cancer cell line by arresting it at the Go/G1 phase of the cell cycle.

## Data Availability

The datasets from the current study are available from the corresponding author on reasonable request.

## Abbreviations

ATCC: American Type Culture Collection
DAPI: 4’,6-diamidino-2-phenylindole
DMSO: dimethyl sulfoxide
E: epanorin
FBS: fetal bovine serum
PBS: phosphate saline buffer
SRB: sulforhodamine-B
TMX: tamoxifen
TdT: terminal deoxynucleotidyl transferase
TRIS: tris(hydroxymethyl)aminomethane
